# A New Uterine Speculum

**Published:** 1874-11

**Authors:** 


*A New Uterine Speculum.*

Dr. Swan M. Burnett gave, last month, under his proper head, a description of a new Speculum, the invention of his fellow-townsman, Dr. R. Knaffl. We are decidedly impressed with the value of the instrument. Dr. Knaffl is having them made under his immediate supervision, and will, he informs us, forward any one the instrument for the price, $12. Address Dr. R. Knaffl, Knoxville, Tennessee. W. T. G.

